# Editorial: Buffalo Genetics and Genomics

**DOI:** 10.3389/fgene.2021.820627

**Published:** 2022-01-28

**Authors:** Hamdy Abdel-Shafy, Tingxian Deng, Yang Zhou, Wai Yee Low, Guohua Hua

**Affiliations:** ^1^ Department of Animal Production, Faculty of Agriculture, Cairo University, Giza, Egypt; ^2^ Key Laboratory of Buffalo Genetics, Breeding and Reproduction Technology, Buffalo Research Institute, Chinese Academy of Agricultural Sciences, Nanning, China; ^3^ Key Laboratory of Agricultural Animal Genetics, Breeding and Reproduction of Ministry of Education, College of Animal Science and Technology, Huazhong Agricultural University, Wuhan, China; ^4^ The Davies Livestock Research Centre, School of Animal and Veterinary Sciences, University of Adelaide, Adelaide, Australia

**Keywords:** buffalo, evolutionary biology, population genetics, molecular genetics, omics

**Editorial on the Research Topic**

**Buffalo Genetics and Genomics**

Buffalo (*Bubalus bubalis*) are important livestock species with significant contribution to food security for thousands of years as a source of milk, meat, leather, dung, hide, horns, traction power, etc. Buffalo production is almost doubled during the last decades due to the improvement in management and nutrition practices along with advanced breeding approaches. To ensure more food security, it is important to sustain the improvement and efficiency of buffalo production to meet the current and upcoming human needs. Genetic improvement is usually used to achieve this goal by selecting the best individuals and breeding them to pass down their favorable genetic materials to the next generations. In this regard, the merit of an animal is predicted in terms of its estimated breeding values (EBVs) even without knowledge of the genetic control of the relevant traits.

With the release of buffalo genome assemblies such as the upgraded reference with long read sequencing (Low et al., 2019), the revolution of high-throughput genotyping technologies has opened the field of buffalo breeding to use omics information to increase the efficiency of selection, including but not limited to genomic prediction, genome-wide association studies (GWASs), evolutionary biology, and functional genomics. These approaches are showed the potential to significantly alter our understanding of the genetic basis of economically important traits in buffalo and enable the scientists to draw a complete picture that previously had major gaps. In this regard, our research topic yielded eleven publications covering diverse approaches and ideas, e.g., classical breeding, genomic prediction, candidate genes, and molecular characterization of different buffalo breeds.

The increased efficiency of production during the last decades is commendable. Although persistency for milk production traits has economic importance, limited studies have been performed so far to determine their genetic parameters in buffalo. Therefore, Nazari et al. estimated the genetic parameters of different persistency measures for milk production traits in Iranian buffalo. They proposed persistency measures of fat production that had favorable low genetic correlations with total milk yield; hence it has an additional benefit when designing breeding schemes. However, the implementation of successful breeding programs based on classical prediction in buffalo is hindered by the lack of sufficient pedigree information traced back many generations ago. This is partially due to natural mating in buffalo, which is still a common reproductive approach used in most farms. A possible solution is to use genetic groups during estimation for variance component and EBV. However, as the percent of missing genealogies increased, the accuracy of prediction is going to decrease regardless the genetic grouping strategies and trait analyzed (Gómez et al.). Another possible solution to overcome the missing pedigree information is to use genomic data. Even with the availability of pedigree information, genomic methods can provide more accurate prediction than those of traditional estimations. For example, the average accuracies for GBLUP and ssGBLUP were increased by 0.03 and 0.08 units over pBLUP (0.21), respectively for milk production traits in Philippine buffalo (Herrera et al.). Although these results are promising, the advantage of using genomic information for genetic improvement in buffalo is still lower than what was expected. It would be attribute to the small number of genotyped animals, using animal own performance, and small sample size (Abdel-Shafy et al., 2020a). One possible solution is to establish a multi-breed reference population (Bolormaa et al., 2013). In this case, it is very important to ensure that the target breed is presented in the multi-breed reference population; otherwise, the accuracy of prediction will be very low due to the inconsistence of linkage disequilibrium (LD) among breeds. In this regard, Rahimmadar, et al. studied the LD structure among different buffalo breeds. They found that the LD measure among SNPs is decreased by increasing the physical distance from 100 Kb to 1 Mb. They also reported that the LD patterns were almost similar among studied breeds. Therefore, the multi-breed reference population for buffalo would be established to increase the accuracy of prediction.

Recently, it has been reported that incorporating biological information and pre-selected genetic markers can increase the accuracy of prediction (Hayes and Daetwyler, 2019). Detection of these loci can be achieved by GWASs, as it has been previously reported for milk production traits in different buffalo breeds (de Camargo et al., 2015; El-Halawany et al., 2017; Iamartino et al., 2017; Mokhber, 2017; da Costa Barros et al., 2018; Liu et al., 2018; Herrera et al., 2018; Lu et al., 2020; Abdel-Shafy et al., 2020b; Awad et al., 2020). However, none of the detected regions was overlapped among different populations and/or validated. In this case, candidate gene approaches would be a complementary method to accurately identify genetic markers and/or causative mutations associated with the relevant trait (Wilkening et al., 2009). In this regard, Tyagi et al. suggested several promising genes for milk production and immunity to be considered for further studies in Indian Murrah buffalo. Likewise, Cosenza et al. and Rehman et al. have intensively studied the evolutionary relationship, comparative genomic, physiochemical properties, and association analysis of casein gene family in different buffalo breeds. They provided useful information about the roles of casein gene family for the variation in milk production traits. In addition, Zhu et al. and Zhang et al. investigated the long non-coding RNAs (lncRNAs) profiles of adipose and muscle tissues in buffalo. They have been identified and verified several differentially expressed lncRNAs in adipose and muscle tissues revealing the importance of lncSAMM50 in lipid accumulation of buffalo adipocytes.

Since cattle and buffalo are closely related species, it is common to compare the findings from buffalo studies with their relevant ones from previous cattle studies. In this regards, Shao et al. compile the genetic parameters and GWASs for different reproductive traits in both cattle and buffalo populations and highlighted possible options to be implemented for improving buffalo breeding. Recently, the research priorities and strategic plans in developing countries have focused on improving the performance of local breeds to face climate change. Swamp buffalo, which are mainly used for agricultural operations in China and Southeast Asian countries, are currently facing additional challenge of being neglected due to rising farm mechanization. This subspecies can be developed for milk and/or meat production under harsh environments and can be used as a strategic option to secure the income of smallholders. Therefore, the challenges and possible opportunities for improving the productivity of swamp buffalo in the Southeastern Asia are comprehensively discussed by Pineda et al.
